# BMAL1 associates with chromosome ends to control rhythms in TERRA and telomeric heterochromatin

**DOI:** 10.1371/journal.pone.0223803

**Published:** 2019-10-21

**Authors:** Jinhee Park, Qiaoqiao Zhu, Emily Mirek, Li Na, Hamidah Raduwan, Tracy G. Anthony, William J. Belden

**Affiliations:** 1 Department of Animal Sciences, Rutgers, The State University of New Jersey, New Brunswick, NJ, United States of America; 2 Department of Nutritional Sciences, Rutgers, The State University of New Jersey, New Brunswick, NJ, United States of America; Karlsruhe Institute of Technology, GERMANY

## Abstract

The circadian clock and aging are intertwined. Disruption to the normal diurnal rhythm accelerates aging and corresponds with telomere shortening. Telomere attrition also correlates with increase cellular senescence and incidence of chronic disease. In this report, we examined diurnal association of White Collar 2 (WC-2) in Neurospora and BMAL1 in zebrafish and mice and found that these circadian transcription factors associate with telomere DNA in a rhythmic fashion. We also identified a circadian rhythm in *Telomeric Repeat-containing RNA* (*TERRA*), a lncRNA transcribed from the telomere. The diurnal rhythm in *TERRA* was lost in the liver of *Bmal1*^-/-^ mice indicating it is a circadian regulated transcript. There was also a BMAL1-dependent rhythm in H3K9me3 at the telomere in zebrafish brain and mouse liver, and this rhythm was lost with increasing age. Taken together, these results provide evidence that BMAL1 plays a direct role in telomere homeostasis by regulating rhythms in *TERRA* and heterochromatin. Loss of these rhythms may contribute to telomere erosion during aging.

## Introduction

Circadian disruption affects a multitude of physiological processes and is implicated in the development of age-related diseases such as metabolic syndrome, cardiovascular disease and cancer [1]. The circadian clock is built upon mechanistically conserved transcriptional feedback loops that generate physiological and behavioral rhythms coinciding with 24 h oscillations in light and dark cycles [2–4]. In vertebrates, the regulatory loop is driven by the transcriptional activators CLOCK and BMAL1, which activates the expression of the negative elements *Period* (*Per1*, *Per2*, and *Per3*) and *Cryptochrome* (*Cry1* and *Cry2*). In Neurospora, a similar feedback loop is controlled by the transcriptional activators White Collar-1 (WC-1) and White Collar-2 (WC-2), which drive expression of the negative element *frequency* (*frq*). The interlocked and cooperative feedback loops of the circadian clock generate rhythms in *clock-controlled genes (ccgs)* to help maintain phase-specific outputs in biological processes [5].

Chromatin remodeling and post-transcriptional modifications to histones are crucial elements in circadian negative feedback, generating rhythms in permissive and repressive chromatin at *ccgs*. In *Neurospora*, *Drosophila* and mammals, the repressive chromatin is composed of histone H3 lysine 9 di- and tri-methylation (H3K9me2/H3K9me3) bound by heterochromatin protein 1 (HP1). The rhythm in facultative heterochromatin also occurs at the *ccg*, Albumin *D-element binding protein* (*Dbp*) [6–8]. In addition, there are diurnal oscillations in H3K9me3 throughout the genome that coincide with age-related changes to diurnal gene expression [9]. In contrast to circadian regulated facultative heterochromatin, which is dynamic, constitutive heterochromatin containing H3K9me3 is largely believed to remain constant and is found at repetitive regions, pericentric heterochromatin, and telomeres [10–12].

Telomeres are specialized protein-DNA complexes positioned at the distal ends of eukaryotic chromosomes and are composed of a TTAGGG repeat bound by shelterin [13–15]. Telomeres are packaged into heterochromatin containing H3K9me3, H4K20me3, H3K27me3 and bound by HP1 [12, 16]. Shelterin and heterochromatin at the telomere help solve the ‘end-protection problem’ by disguising, capping, and silencing the chromosome ends [17, 18]. However, telomeres are not entirely transcriptionally silent and there is a long non-coding RNA called Telomeric Repeat-containing RNA (*TERRA*) [19]. *TERRA* function is largely inferred because generating a loss-of-function *TERRA* model has proven difficult. *TERRA* is implicated in telomere protection by recruiting factors such as histone H3 lysine 9 methyltransferase (KMT1/Suv39h) and HP1 to promote heterochromatin formation at telomeres [20–22]. Other studies suggest *TERRA* is involved in telomere elongation [23] and replication [24] by telomerase. Disruptions to TERRA can directly or indirectly induce diseases such as astrocytoma and cause accelerated aging or premature cell senescence [25–28].

Despite a strong understanding of the core circadian clock mechanism(s), why and how the clock changes with age and how disruption to the clock impacts aging are existing questions left unanswered. In this report, we reveal that BMAL1 in zebrafish and mice, and WC-2 in *Neurospora*, are localized to the telomere. We also identify a diurnal rhythm in *TERRA* expression and H3K9me3 at telomeres which is lost in *Bmal1*^-/-^ organisms, indicating the rhythm in both is under circadian control. Lastly, we report that *TERRA* and H3K9me3 oscillations at the telomeres decay with age. These findings reveal a direct role for the circadian clock in telomere homeostasis whereby the clock regulates rhythms in *TERRA* and heterochromatin. These data provide valuable insight into the mechanisms underlying the advanced aging phenotypes observed with circadian disruption.

## Materials and methods

### Animal care and Neurospora growth

Neurospora conidia were suspended in liquid culture medium (LCM) containing 2% glucose (1x Vogel’s salts, 2% glucose, 0.17% arginine) and grown in 100 mm Petri dishes overnight at 30°C to generate mycelia mats. Plugs were cut and used to inoculate flasks containing 100 ml of LCM and grown at 25°C for 2 d. For circadian time course experiments, strains were entrained with a standard light to dark transfer and harvested after a timed incubation in the dark (4, 8, 12, 16, 20, 24, 28, and 32 h). Tissue was crosslinked with 1% formaldehyde, quenched with 100 mM glycine, harvested by filtration, frozen in liquid nitrogen, and then ground with a mortar and pestle in the presence of liquid nitrogen.

All animal experiments were approved by the Institutional Animal Care and Use Committee (IACUC) of Rutgers University. Wild-type zebrafish were purchased from ZIRC (Zebrafish International Resource Center, Oregon) and housed according to procedures approved by the Policy on Housing of Vertebrate Animals Outside of Animal Facilities. Fish were fed twice daily and maintained under a 14-hour light: 10-hour dark cycle for breeding, or 12-hour:12-hour light:dark cycle for diurnal entrainment. Adult fish were kept in system water (conductivity 800 ± 200 μS and pH 7.5). Embryos and young larvae were maintained in egg water (30 mg/l Instant ocean in deionized water). Fish were sacrificed by emersion in cold MS-222 (300 mg/l, Sigma) and dissected under PBS.

To perform experiments in the absence of BMAL1, male and female C57BL/6J mice (8–12 wk old) carrying a homozygous deletion of *Bmal1* (B6.129-*Arntl*^*tm1Bra*^/J, Jackson Laboratories) were used alongside wild-type controls [29]. For the genotyping, ear tissues from 8 wk-old mice bred in-house at the Bartlett animal facility were collected, and genomic DNA was extracted. PCR-based genotyping was performed as described as described [29], and male and female WT and homozygous *Bmal1*^*-/-*^ mice were sorted and used for experiments. For time-course sampling, mice were maintained under a 12-hour:12-hour light-dark cycle and sacrificed at 4-hour intervals. All relevant data was analyzed for rhythms using Cosinor with DiscoRhythm [30].

### Antibodies

A collection of custom-made and commercial rabbit polyclonal antibodies was used to ensure scientific rigor and rule out potential artifacts due to non-specific binding common to antibody preparations. BMAL1 antibodies that recognize the mouse isoform were purchased from Abcam (ab3350). We also generated a BMAL1 antibody that recognizes the zebrafish and mice isoforms. Briefly, three custom peptides corresponding to the proposed surface-exposed regions of BMAL1 (Peptide #1 CSPGGKKIQNGGTPD, #2 CSSSDTAPRERLIDA, #3 CSTNCYKFKIKDGSF) were combined and used as the immunogen. The antibodies were tested by western on whole tissue isolated from zebrafish or mice (S1 Fig). For the ChIP experiments, the antibodies generated in this report were further affinity purified using the 3 peptides and Sulfolink immobilization kit (Thermo scientific, 44995) following manufacturer's guidelines. The 3 peptides were resuspended in 2 mercaptoethylamine-HCl (2-MEA) solution at 37°C for 1.5 hours. The reduced peptides were mixed with the SulfoLink resin and coupled to the beads by rocking for 15 min and then allowed to settle for 30 min at room temperature. The protein concentration of the flow-through and the unbound fraction was compared to determine the coupling efficiency. After coupling, the crude sera were loaded into the SulfoLink Column for affinity purification. Antibody bound to resin was washed three times with Tris-buffered saline and eluted in 0.1M glycine-HCl (pH 2.5). Elutes were neutralized by adding 1:20 the volume of 1M Tris-HCl (pH8.5). Antibodies specific to H3K9me3 were purchased from Abcam (Abcam, ab8898).

### Chromatin immunoprecipitation (ChIP)

ChIP experiments followed the general procedure described previously [31] but modified for zebrafish and mouse tissue as follows. Isolated zebrafish tissue was cross-linked with 1% formaldehyde for 10 min at room temperature then quenched with 0.1M Glycine for an additional 10 min. The cross-linked tissue was snap-frozen in liquid nitrogen and stored at -80°C. The tissue was homogenized with a micropestle in the presence of 100 μl ChIP lysis buffer [0.05 M Hepes (pH 7.4), 0.15 M NaCl, 0.001 μM EDTA, 1% Triton X-100, 0.1% Deoxycholic acid, 0.1% SDS] containing protease inhibitors (2.0 μg/ml leupeptin, 2.0 μg/ml pepstatin A, 1.0 mM PMSF). Additional cell disruption and crude chromatin shearing were achieved by sonication at low power (2 × 20 sec at 10% power using a cup sonicator). Lysates were transferred into polystyrene sonication tubes and sonicated again (6 × 20 s at 20% power). The resulting lysates were cleared of cellular debris by centrifugation at 5000 × *g* for 10 min. The sonication regime consistently yielded chromatin sheared to an average size of 500 bp. The WC-2 ChIP has been described previously [32]. For the BMAL1 ChIP, we used approximately 2.0 mg of sheared chromatin and 200 μg for the H3K9me3 ChIP. Prior to the ChIP, the BMAL1 or H3K9me3 antibodies were prebound to protein A-conjugated magnetic beads (Dynabeads). The ChIP was washed five times with RIPA buffer and then eluted twice with 0.1 M sodium bicarbonate, 1.0% SDS for 10 min at 37°C. The cross-links were reversed by adding 2 μl of 5M NaCl and incubated for a minimum of 4 hours at 65°C. Protein was removed by the addition of 1 μl of proteinase K (10 mg/ml), 4 μl of 1.0M Tris-HCl (pH 6.5), 2 μl of 0.5M EDTA(pH 8.0), and incubated at 42°C for 1-hour. DNA was purified by a phenol/chloroform extraction. The relative levels of BMAL1 or H3K9me3 at *Per2* E-box and telomere were determined by qPCR. All the oligonucleotides used in the report are contained in S1 Table.

H3K9me3 ChIP from mouse liver were also performed on enriched nuclei preparations. Tissue was harvested and chopped into small pieces on the ice and cross-linked with 1% formaldehyde for 10 min, then quenched with 0.1M glycine for 10 min at room temperature. Nuclei were prepared from the cross-linked liver tissue as follows. Tissue was homogenized with a micropestle in 1 ml of ice-cold buffer A [250 mM sucrose, 5 mM MgCl_2_, and 10 mM Tris–HCl (pH 7.4)]. The crude lysate was centrifuged at 600 x *g* for 10 min at 4°C to pellet the nuclei. The supernatant was discarded, and the nuclei were washed with 1ml of ice-cold buffer A and centrifuged again at 600 x *g* for 10 min at 4°C. The crude nuclei pellet was resuspended in 1 ml of ice-cold buffer B [2.0 M sucrose, 1 mM MgCl_2_, and 10 mM Tris–HCl (pH 7.4)], mixed and centrifuged at 16,000 x *g* at 4°C for 30 min. The tube was inverted and pushed gently against a paper towel, removing most of the upper layer. The nuclei were resuspended in 30 μl ChIP lysis buffer containing protease inhibitors and transferred to a polystyrene tube and sonicated 8 × 30 s at 20% power. The resulting lysates were cleared of cellular debris by centrifugation at 13,000 x *g*. 200 μg of sheared extract was used for BMAL1 ChIP and 100 μg of for H3K9me3 ChIP. For each sample, 3 μl of antibody was pre-bound to 30 μl of magnetic beads overnight.

### Northern and slot blot

Total RNA from zebrafish tissues was isolated by Trizol (Invitrogen) following the manufacturer's protocol. 3–5 μg of total RNA were incubated for 15 min at 65°C in RNA loading buffer (1X MOPS, 56.8% formamide, 20.4% formaldehyde, 11% RNA loading dye [1mM EDTA pH8, 0.23% bromophenol blue, 50% glycerol]) and resolved by electrophoresis on 1.2% agarose gel containing 5% formaldehyde for 3 hours at 70 V in 1X MOPS buffer (2 mM EDTA, 20 mM MOPS 5 mM sodium acetate). Gels were rinsed two times with distilled water then soaked in 10 X SSC for 30 min then transferred to a hybond N^+^ membrane by capillary transfer. RNA was UV-crosslinked to the membrane and hybridized with DIG-labeled TERRA specific probe at 65°C overnight. Membranes were washed with 2X SSC, 0.1% SDS at 42°C two times and 0.1% SSC, 0.1% SDS at 65°C three times. The membrane was then incubated in DIG blocking buffer for 2-hours followed by incubation with anti-digoxigenin Fab fragments for 30 min. The membranes were washed with 1X maleic acid, 0.3% tween 5 times for 10 min and visualized using CDP-star (Roche). The telomere probe was generated as follows; pSXneo279 (T2AG3) was obtained from Addgene (plasmid #12403) and used as a template to amplify a fragment containing TTAGGG repeats. The PCR amplified TTAGGG product was cloned into pCR4-TOPO vector (Invitrogen). After sequencing, one clone contained 35 TTAGGG repeats was selected and used with DIG Probe Synthesis Kit (Roche Diagnostics) with pTelo250F and pTelo250R oligos.

For the telomere slot blot, ChIP DNA was prepared in 300 μl of denaturation solution (0.4 M NaOH, 10 mM EDTA) then boiled at 95°C for 10 min and spotted on the hybond N^+^ membrane under a vacuum. Membranes were pre-hybridized in DIG easy hyb (Roche) for 2 h and then hybridized overnight with a DIG-labeled oligonucleotide probe specific to the telomere (TTAGGG)_5_.

### Analysis of ChIP-Seq and RNA-Seq

Published WC-2 ChIP-seq [33] and BMAL1-ChIP seq data [34, 35] were downloaded and mapped to the corresponding reference genome, (NC10 or mm10) using Burrows-Wheeler Aligner [36]. Visualization of binding to the telomere was done using Integrative Genomics Viewer (IGV) [37].

## Results

### Circadian transcription factors localize to the telomere

**Fig 1:**
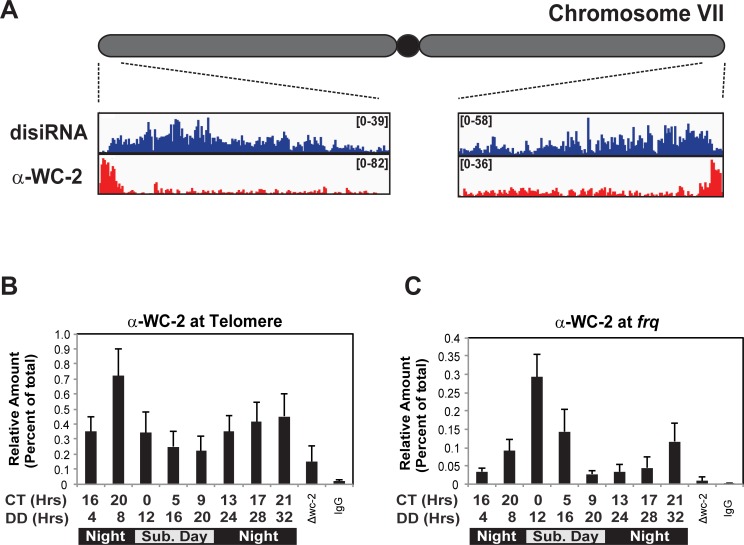
WC-2 is associated with the telomeres in *Neurospora*. (A) WC-2 ChIP-seq and Argonaute-associated RIP-seq data in Neurospora showing WC-2 is localized to telomeres, and the binding is adjacent to Argonaute-associated Dicer-independent small interfering RNAs (disiRNA). Data were mapped to the *Neurospora* genome and visualized with Integrative Genomics Viewer (IGV). The interaction between WC-2 and telomere was confirmed by ChIP-qPCR under circadian entrainment (B) using the *frq* C-box as a control (C). The data were obtained from 4 biological replicates, error bars represent the SEM and Cosinor analysis gave p-value < 0.01, q-value < 0.01.

While examining published WC-2 ChIP followed by DNA sequencing (ChIP-seq) data, we observed that WC-2 appeared to localize to telomeres and localization was adjacent to Dicer-independent small interfering RNAs (disiRNA) (Fig 1A) [33, 38]. To confirm this observation, we performed ChIP-qPCR on circadian entrained cultures with a WC-2 antibody and oligonucleotides adjacent to the telomere repeat using *Δwc-2* as a control. The results confirmed association and revealed a rhythm in WC-2 association with the telomeres (Fig 1B). Moreover, the profile of WC-2 binding at the telomeres appeared analogous to WC-2 association at the C-box in the *frequency* (*frq*) promoter (Fig 1C). The identification of WC-2 at telomeres suggests the circadian clock may play a direct role in telomere regulation. To bolster these observations, we proceeded to explore binding in tissues from zebrafish and mice to establish conservation in higher order species.

**Fig 2:**
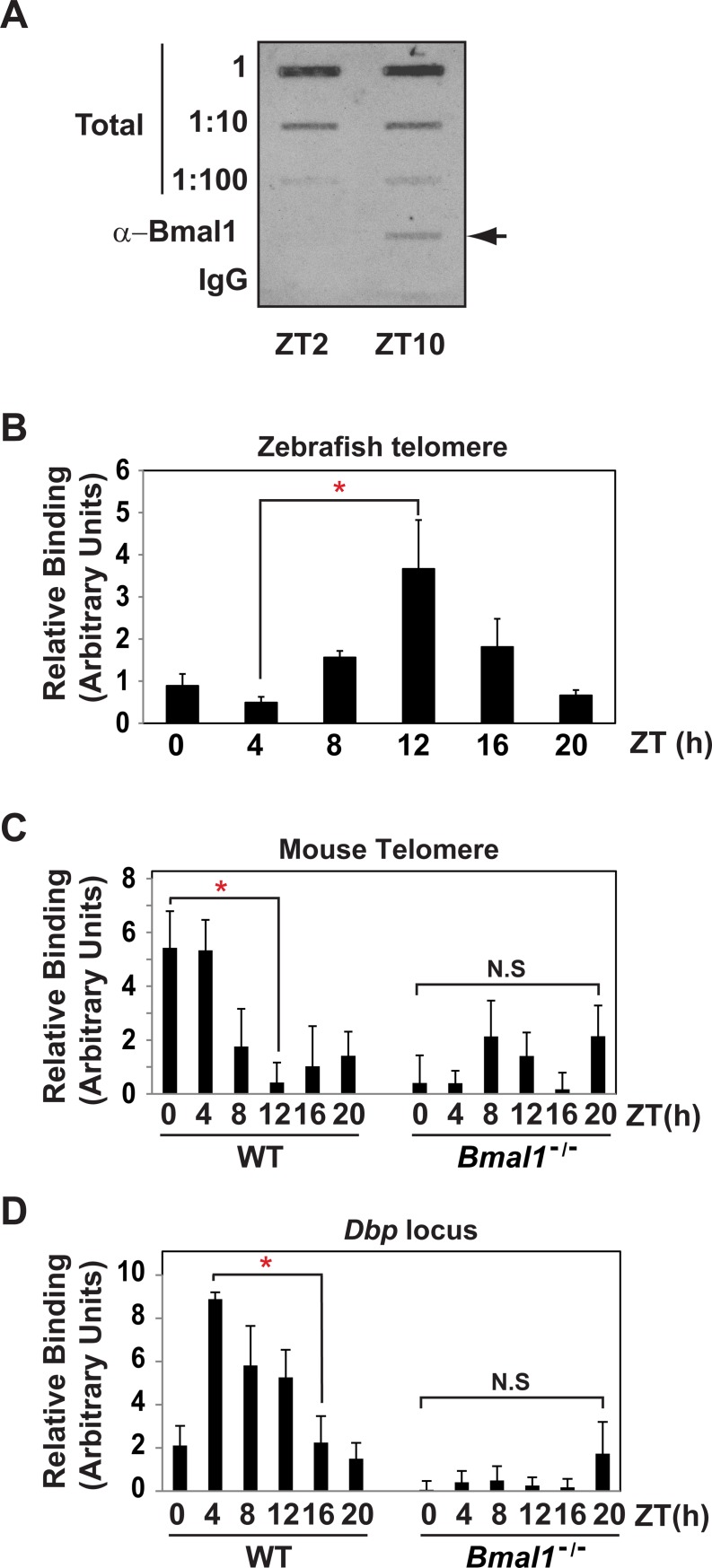
BMAL1 at associates with the telomere in zebrafish and mice. (A) Bmal1 ChIP-slot-blot from zebrafish skeletal muscle using the telomere repeat (TTAGGG)_5_ probe. (B) Bmal1 ChIP-qPCR over full circadian time course from zebrafish brains isolated every 4 h. The data are from 5 biological replicates, error bars represent the SEM and Cosinor analysis gave p-value < 0.005, q-value < 0.01. (C) ChIP examining BMAL1 binding to the telomere on chromosome 13 in WT compared to *Bmal1*^-/-^ mice. (D) Same as in C except binding was assayed for *Dbp*. The data in C and D are from 3 biological replicates and 2 technical replicates. The error bars show SEM and Cosinor analysis yeilded p-value < 0.005 and q-value < 0.005 for WT and p-value > 0.5 and q-value > 0.5 for *Bmal1*^-/-^. Statistical analysis of peak to trough was by one-way ANOVA with Bonferroni Post Hoc test gave p-value < 0.05).

As a first step in determining whether circadian transcription factor binding to the telomere was fungal-specific or a general clock mechanism conserved in higher order species, we examined BMAL1 ChIP-seq data (GS26602, GSE39977) [34, 35]. Inspection of telomere sequence in *mus musculus* mm10 genome indicated BMAL1 was enriched at the telomere (S2 Fig). To conclusively determine if BMAL1 localized to the telomeres and rule out potential ChIP-seq artifacts, we examined the association of Bmal1 with the telomere by ChIP-slot blot on zebrafish brain tissue using the telomeric repeat (TTAGGG) as a probe at ZT2 and ZT10. The assay showed that Bmal1 associates with telomere DNA and binding is higher at ZT10 relative to ZT2 (Fig 2A). Next, we examined Bmal1 binding by ChIP-qPCR [39] in zebrafish brain over a full 24-h cycle sampling every 4 h and found a diurnal rhythm that peaked around ZT12 (Fig 2B).

Rhythmic binding of BMAL1 to the telomere may have major physiological consequences. Therefore, to conclusively determine if BMAL1 associates with the telomere, we tested binding in mice using the *Bmal1* knockout (*Bmal1*^-/-^) as a negative control. The BMAL1 ChIP was performed in WT and *Bmal1*^-/-^ mouse livers over a 24-hr diurnal cycle. As a control for entrainment and rhythmicity, we measured the diurnal expression of *Per2* and *Bmal1* by RT-qPCR. Expression of *Per2* and *Bmal1* gave the expected phasic-specific pattern in WT, which was absent in *Bmal1*^-/-^ (S3 Fig). Next, we tested whether BMAL1 localizes to the telomere by measuring the interaction between BMAL1 and the telomere and found a rhythm that peaked between ZT0—ZT4 in WT (Fig 2C). Analysis of peak to trough levels (ZT0 vs. ZT12) indicated a significant change in BMAL1 binding and Cosinor analysis revealed it was rhythmic, whereas the background amplicons in *Bmal1*^-/-^ mice showed no significant difference among any time points or with the trough in WT (ZT12) (Fig 2C). As a further control for the BMAL1 ChIP, we examined BMAL1 localization to albumin *D element-binding protein* (*Dbp*), a known clock-controlled gene with E-box element in its promoter. We found a similar, albeit slightly phase delayed diurnal interaction consistent with previous reports (Fig 2D) [40].

### *TERRA* is diurnally regulated

**Fig 3:**
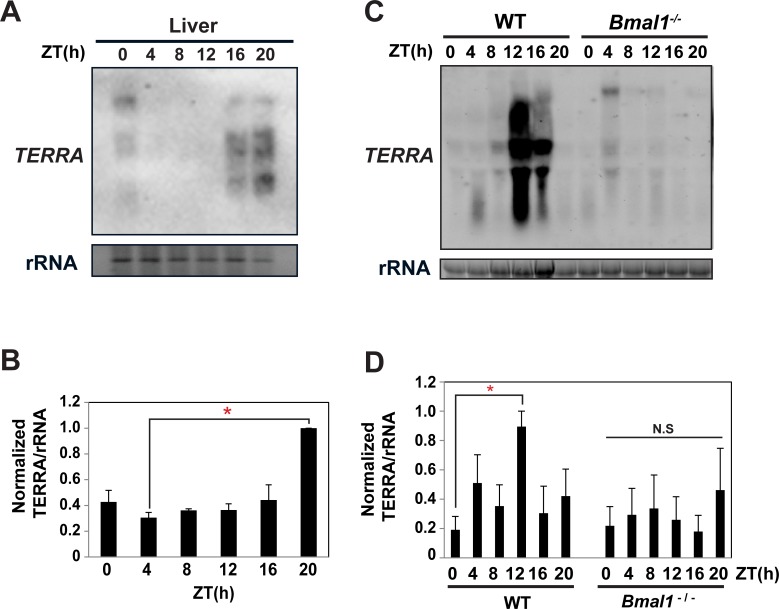
Identification of diurnal rhythm in *TERRA*. (A) A representative *TERRA* Northern blot from zebrafish over a full diurnal time course. (B) Quantification of *TERRA* Northern blots normalized to rRNA from three independent biological replicates. Cosinor analysis yielded p-value < 0.01 and q-value < 0.01 (C) A representative *TERRA* Northern blot performed on total RNA isolated from WT and *Bmal1*^-/-^ mice over a full-time course. (D) The quantification of 3 independent Northern blots normalized to rRNA. The error bars in B and D represent the SEM. Cosinor analysis yielded p-value < 0.05 and q-value < 0.05. Statistical analysis of peak to trough was done by one-way ANOVA and Bonferroni Post Hoc test (*; p ≤ 0.05). In all instances *TERRA* fell in the expected size range between 100 nt and 9 kb as represented by the smear [19].

The association of clock transcription factors with the telomere in *Neurospora*, zebrafish, and mice led to the obvious question of BMAL1 function at the telomere. Thus, we sought to determine if *TERRA* expression is rhythmic and whether its expression was dependent on BMAL1. *TERRA* is a heterogenous and presumably unstable transcript containing the UUAGGG repeat that ranges in size between 9.0kb and 100 nucleotides (nt) and ultimately get processed in the Dicer independent siRNAs called tel-sRNA [19, 41]. The heterogeneity of *TERRA* causes it to appear as a smear on a Northern blot [19]. As an initial test, we examined *TERRA* in zebrafish at 2 diurnal time points (ZT2 and ZT10). Northern blot on total RNA using a *TERRA*-specific oligonucleotide probe (TTAGGG)_5_ revealed *TERRA* was higher at ZT2 relative to ZT10 in both brain and liver (S4 Fig). Next, we examined *TERRA* expression over a 24-h cycle sampling at 4-h intervals. A representative Northern blot from three independent biological replicates indicates there is a rhythm in *TERRA* expression in liver with a peak at ZT16-ZT0 and trough between ZT4-ZT12 (Fig 3A). Quantification and statistical analysis of the 3 independent biological replicates confirmed the rhythm peaked during the night (Fig 3B).

We proceeded to examine if the diurnal rhythm in *TERRA* was dependent on BMAL1. To accomplish this, we performed *TERRA* Northern blots on WT and *Bmal1*^-/-^ mouse liver over a 24-hr cycle. The *TERRA* Northern revealed a diurnal rhythm in the liver of WT mice that was absent in *Bmal1*^*-/-*^ mice (Fig 3C). Quantification of the three independent biological replicates shows that TERRA peaked at the light to dark transition (Fig 3D, and S5 Fig). Zebrafish are diurnal while mice are nocturnal and the rhythm in *TERRA* in both systems was consistent with the peak in expression occurring just before their respective activity cycles. We also examined *TERRA* in entrained human osteosarcoma U2OS cells, which is a widely used cell line that has alternative lengthening of telomeres (ALT) and is an established model for circadian research. U2OS cells showed a low amplitude rhythm in TERRA which peaked at ZT16 when normalized to rRNA (S6 Fig).

### Diurnal regulation of heterochromatin at telomere

**Fig 4:**
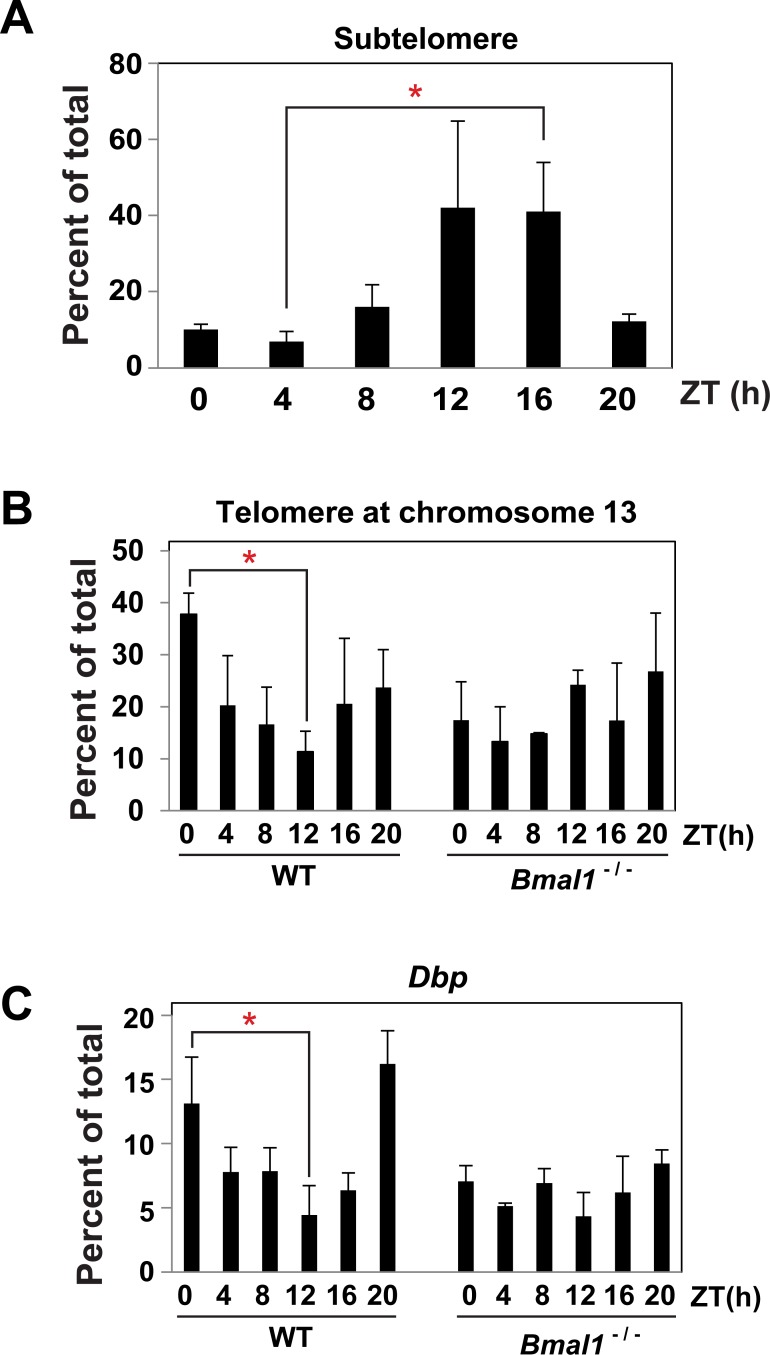
Rhythms in heterochromatin at telomere in zebrafish and mice. (A) H3K9me3 ChIP at telomere region of chromosome 1 in zebrafish brain tissue. Level of H3K9me3 was determined by qPCR using oligonucleotides in S1 Table. (B) H3K9me3 at the telomere at chromosome 13 were determined by ChIP from mouse liver in WT and *Bmal1*^-/-^. (C) Same as in B except oligonucleotides were specific to *Dbp* locus. The data are averages from a minimum of 4 independent biological replicates. Error bars represent the SEM. Cosinor analysis yeilded p-value < 0.05 and q-value < 0.05 for WT animals (Zebrafish and mice) but p-value > 0.5 and q-value > 0.5 for *Bmal1*^*-/-*^. Statistical analysis shon was by one-way ANOVA of peak to trough with Bonferroni Post Hoc test (*; p ≤ 0.05).

One proposed function of *TERRA* is to guide heterochromatin. Therefore, we sought to determine if the rhythm in *TERRA* was accompanied by a rhythm in heterochromatin; similar to clock genes. We conducted H3K9me3 ChIP on cross-linked zebrafish brain tissue harvested every 4-hour for 24-hours and this revealed a rhythm in H3K9me3 that peaked around ZT12 to ZT16 (Fig 4A). Next, we tested whether the H3K9me3 rhythm was conserved in mice and dependent on BMAL1. H3K9me3 ChIP in the livers of WT and *Bmal1*^-/-^ mice indicated there was a rhythm in H3K9me3 at the telomere in WT but absent in *Bmal1*^-/-^ (Fig 4B), indicating telomere heterochromatin is circadian-regulated and required BMAL1. We also measured H3K9me3 at *Dbp* in WT versus *Bmal1*^-/-^ as a positive control and to determine if the H3K9me3 rhythm at DBP was also dependent on *Bmal1*^-/-^ (Fig 4C) [40]. Consistent with previous findings, we observed an H3K9me3 rhythm at *Dbp* in WT liver and we now show that this rhythm is dependent on BMAL1 indicating the rhythm in H3K9me3 requires a functional circadian oscillator.

**Fig 5:**
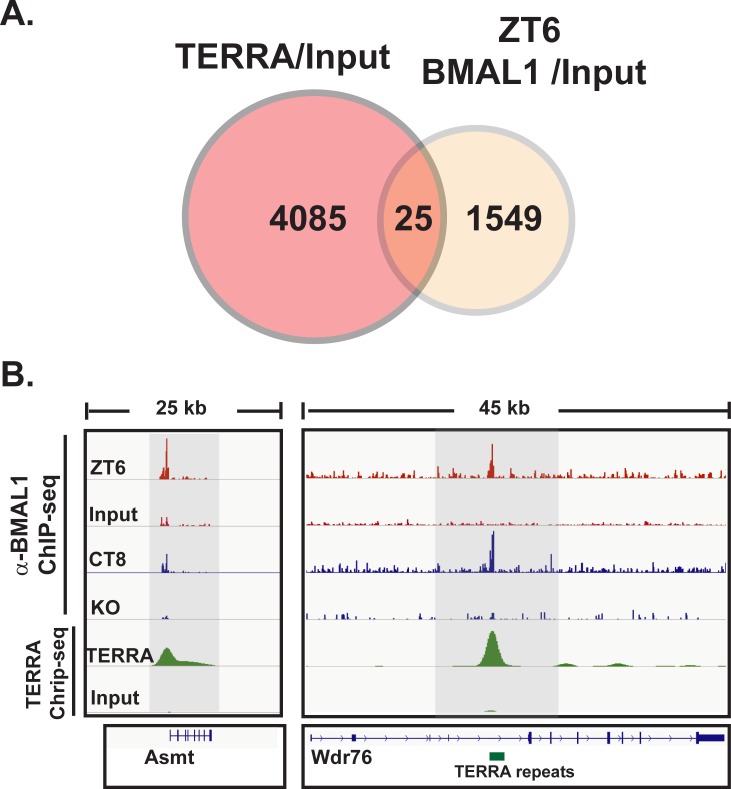
TERRA co-localizes with BMAL at circadian-regulated genes. (A) Overlap of *TERRA* and BMAL1 at loci throughtout the genome. *TERRA* CHIRT-seq (GSE79180) and BMAL1 ChIP-seq (GSE26602 and GSE39977) were downloaded from GEO and the extent of overlap was determined by Venn analysis. (B & C) Locus-specific view showing co-localization of *TERRA* and BMAL1 at two circadian-regulated genes (*Asmt* and *Wdr76*).

Recent advancements in understanding *TERRA* revealed its localization is not restricted to telomeres. Instead, *TERRA* is found at over 4000 genomic locations containing the telomere repeat sequence and appears to sequester the chromatin-remodeling enzyme, ATRX [20]. Our findings that BMAL1 associates with the telomere repeat and the rhythm in *TERRA* requires BMAL1 led us to explore other possible connections between the circadian clock and telomere rhythms. Therefore, we examined potential overlap between BMAL1 and *TERRA* localization. We determined there were 25 genomic loci where *TERRA* and BMAL1 overlap (Fig 5A). Not surprisingly, analysis of the 25 genes using CircaDB [42] indicated all were circadian regulated genes. For example, one of the loci, *Asmt*, which encodes acetylserotonin methyltransferase, is important for circadian physiology and regulates melatonin synthesis while another, *Wdr76* encodes a component of the PER complex (Fig 5B and 5C). Of note, all 25 contain the telomere repeat as a non-canonical E-box.

### Aging and stress affect diurnal regulation of TERRA and heterochromatin

**Fig 6:**
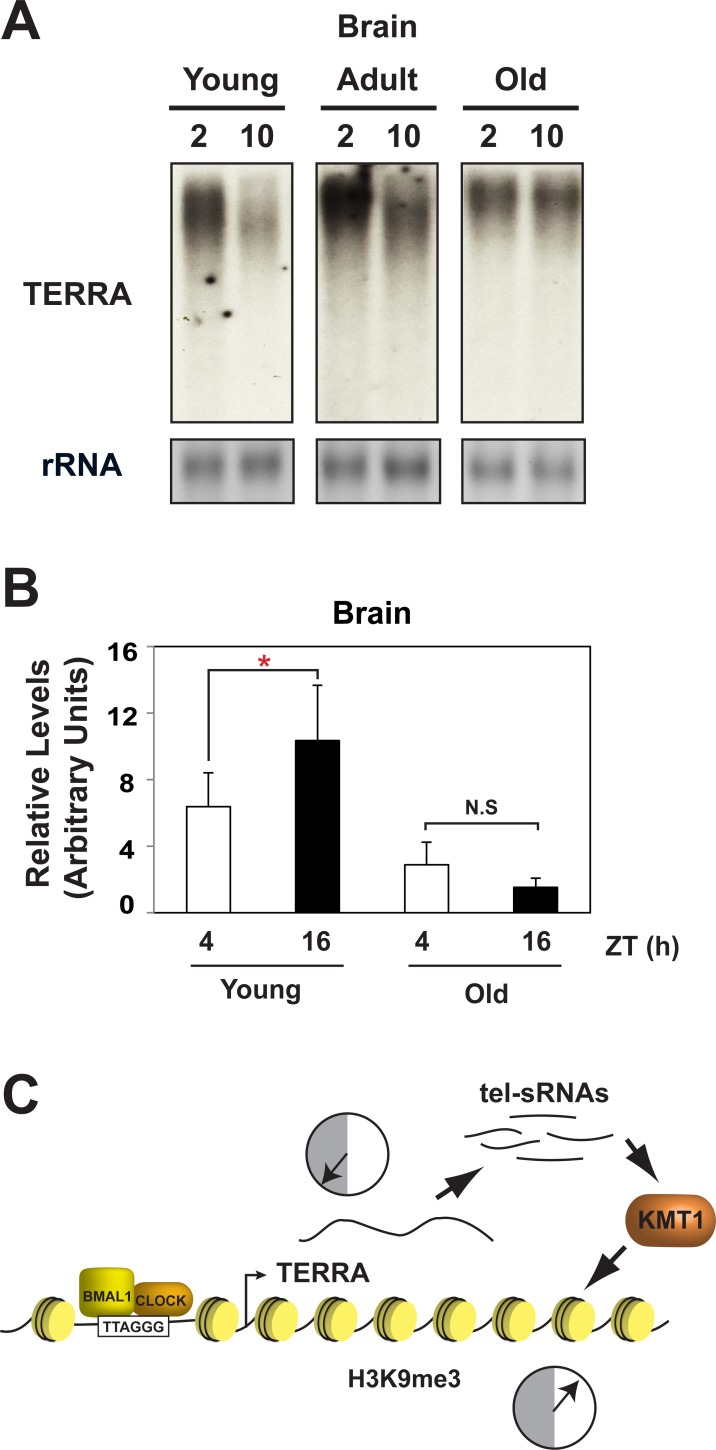
Aging alters the diurnal rhythms in *TERRA* expression and H3K9me3. (A) A representative Northern blot examining diurnal *TERRA* expression between ZT2 and T10 at different ages and conditions (Young: 4M, Adult: 12M, Old: 20M, and Stressed). (B) Level of H3K9me3 at the telomere region of chromosome 1 was measured by ChIP in zebrafish brain corresponding to different ages (Young; 4M, Old; 20M). The experiment is from 3 independent biological replicates. The error bars represent the SEM. Analysis was by t-test and the asterisk indicates *p* ≤ 0.05. (C) Potential model of circadian regulation at the telomeres. BMAL1 (and possibly CLOCK) associate with the telomere repeat and direct rhythms in *TERRA* expression. *TERRA* gives rise to tel-sRNAs which guide H3K9me3 of telomere chromatin.

Telomere length is associated with aging and shortened telomeres correlate with increased cellular senescence [43, 44]. Therefore, we sought to gain insight into how and if the diurnal rhythm in *TERRA* changes with age in zebrafish. We tested *TERRA* expression at 3 different ages. In young and adult fish, *TERRA* maintained a rhythm; however, the rhythm in *TERRA* appeared to be muted in the brain of old zebrafish (Fig 6A). Moreover, the diurnal rhythm of H3K9me3 in young fish disappeared in old animals (Fig 6B). Consistent with these findings, we also found age-related loss in *TERRA* and H3K9me3 in liver (S7 Fig). These data support the idea that the rhythm in *TERRA* and H3K9me3 may be blunted or lost with age.

## Discussion

In this report, we find that circadian clock transcription factors (BMAL1 in mammals and zebrafish, and WC-2 in Neurospora) are associated with telomeres and there is a rhythm in binding. We also find diurnal rhythms in other facets of telomere biology. For example, we identified a rhythm in both *TERRA* expression and heterochromatin formation. Of note, these rhythms require a functional circadian oscillator because they are lost in the absence of BMAL1. These observations help to solve an important unexplained paradox in telomere biology; How is *TERRA* expressed when the telomeres are packaged in heterochromatin, which by all accounts should be silent? The answer, explained by these results, is that a phase-specific rhythm in both *TERRA* and heterochromatin enables expression. In other words, when the level of heterochromatin is low, *TERRA* expression is high and vice versa. Ironically, this same phenomenon occurs at the central clock genes where there are anti-phasic rhythms in clock gene expression and heterochromatin formation in *Neurospora*, *Drosophila* and mammals, and this is a key facet in circadian clock regulated gene expression. Furthermore, these data shed light on a previously undiscovered direction connection between the clock and aging which may hold significant clinical applications (see below).

The similarities between core clock gene regulation and telomeres homeostasis can potentially be extrapolated into a unifying model (Fig 6C). In Neurospora, convergent transcription of the *frq*:*qrf* sense:antisense pair gives rise to Argonaute-associated Dicer-independent siRNAs (disiRNA) necessary for DNA methyaltion and heterochromatin [45, 46]. Neurospora disiRNA are also found at telomeres, adjacent to WC-2 (Fig 1A). In mammals, *TERRA* is processed into Dicer-independent telomere-specific small RNAs called tel-sRNAs. These tel-sRNAs are are proposed to play a role in establishing and/or maintaining heterochromatin at the telomere [41]. Based on these similarities, and the rhythm in *TERRA* and heterochromatin, it is interesting to speculate that circadian regulation of *TERRA* may serve to mediate circadian heterochromatin via tel-sRNAs in a similar mechanism to what occurs at *frq* in *Neurospora* and possibly *Per2* in mammals. Support for this comes from a rich supply of literature indicating lncRNAs are invloved in establishing hetertochromatin in imprinting, X-chromosome inactivation, and RNAi-mediated heterochromatin. Thus it seems reasonable to speculate the rhythm in *TERRA* is directly involved in mediating rhythms in telomere heterochromatin [45, 47, 48]. However, there remains a significant amount research to understand the mechanism and timing of this model. For example, an unanswered question in this model is whether the rhythm in heterochromatin is mediated by the PER complex. Consider for a moment that KMT1/SUV39 is a component of the PER complex and there are rhythms in facultative heterochromatin at *Per2* [7, 8]. Then one might envision a mechanism whereby tel-sRNAs guide the PER complex to the telomere to help establish telomere heterochromatin. Supporting this idea, *TERRA* is found associated with proteins that are also associated with PER2, such as NONO and DHX9 in mouse ES cells [20]. Thus, the idea that components of the PER2 complex associates with tel-sRNAs is worthy of further consideration.

Another aspect of this work that bears mentioning is the age-related decline in *TERRA* and heterochromatin rhythm. Clock mutant animal models have many age-related phenotypes indicating the circadian oscillator plays an important role in counteracting aging [49–53]. In addition, telomere length has a rich history of being implicated in aging. Based on this, we did some exploratory analysis on age-related changes to the diurnal rhythm in *TERRA* and heterochromatin and found the rhythm in both is comprimized with age. Taken together, these results suggest that the circadian clock has an essential role in telomere homeostasis by regulating telomeric lncRNA and heterochromatin at the telomere. Moreover, there are other direct connections between circadian clock and telomere homeostasis [54, 55]. The Telomere reverse transcriptase, TERT and its activity were found to be under circadian control [55] and reconstitution of TERT in senescent fibroblasts is necessary for circadian entrainment [54].

One semi-perplexing issue throughout the course of these experiments was the idea that circadian transcription factors bind the telomere repeat (GGGTTA), which differs from the canonical E-box sequence (CACGTG). However, the bHLH domains of CLOCK and BMAL1 prefer a non-canonical 7 bp E-box sequences (AACGTGA or CATGTGA). When viewed in this contect, there is a significant amount of identity between the non-canonical E-box relative to the telomere repeat sequence (AACGTGA vs. AGGGTTA). Furthermore, we also observed that BMAL1 is found at known *ccgs* that overlap at TERRA/telomere repeat sites throughout the genome (Fig 5). So it appears that BMAL1 can use the telomere repeat as a non-caononical E-box. Whether or not BMAL1 is partnered with CLOCK or NPAS2 or another transcirption factors remains unresolved. Another unresolved issue was the phasing of WC-2 and BMAL1 binding telomeres in *Neurospora* and mice. There always appeared to be a slight phase advance in bind relative to clock genes or *Dbp*. It is unclear why this occurred but may be that *TERRA* functions like a circadian lncRNA that is slightly phase advanced.

In conclusion, it is clear there is circadian regulation at telomeres, including a rhythm in *TERRA* and heterochromatin formation, and this has important clinical implications because shortened telomeres correlate with many human diseases. The data presented in this study suggest that alterations to the normal diurnal regulation of telomere homeostasis likely leads to increased senescence when normal circadian rhythms are disrupted and likely impacts genome integrity as well. Whether this mechanism is a driver connecting circadian dysregulation in shift-workers and the higher incidence of circadian and age-associated diseases remains an open question.

## Supporting information

S1 Table Oligonucleotides used in the report. (DOCX)

S1 Fig Test of Bmal1 antibody. **(A)** The Bmal1 antibody was tested for its ability to detect zebrafish Bmal1a and/or Bmal1b. A single band corresponding to the size of Bmal1a was detected and found to be rhythmic in zebrafish tissue (3 independent westerns are shown). **(B)** We also tested whether the antibody could also detect the mouse isoform and compared NIH3T3 cell lysate to *Bmal1*^*-/-*^ tissue lysate and found good specificity. Note that in all Bmal1/BMAL1 ChIPs in this report were confirmed and combined with data generated using the Abcam antibody enhancing the rigor and reproducibility. We also tried the Bethyl BMAL1 antibody but found it to be less satisfactory.
(EPS)

S2 Fig BMAL1 at associates with the telomere in mice. (A) BMAL1-ChIP seq (GSE26602 and GSE39977) indicates BMAL1 binding to the telomere repeat on mice chromosome 17. Zoomed image shows the BMAL1 peaks at the telomere repeat.
(EPS)

S3 Fig The level of transcripts of core clock genes in mice. RT-PCR of *Per2* (A) and *Bmal1* (B) was performed on mouse liver tissue sampled every 4 h. to confirm the rhythmicity. Samples from these same animals were used to measure *TERRA* expression and a portion of the same tissue was crosslinked and used for ChIP.
(EPS)

S4 Fig (A) Northern blot of total RNA isolated from zebrafish brain and liver were probed for TERRA at ZT2 and ZT10.
(EPS)

S5 Fig The diurnal rhythms in mouse *TERRA*. An additional *TERRA* Northern blot done on RNA isolated from WT and *Bmal1*^*-/-*^ mouse liver tissue. This datum was combined with additional blots and Fig 3B, to obtain the quantification shown in Fig 3D.
(EPS)

S6 Fig TERRA appears rhythmic in U2OS cells. (A) Northern blots examining the level of *TERRA* transcript in human osteosarcoma cell line (U2OS) displayed for three independent biological replicates. (B) Quantification of the Northern blots from A was averaged and shown as a bar graph. Error bars show SEM and Analysis was by one-way ANOVA followed by Bonferroni post hoc test (*; p ≤ 0.05).
(EPS)

S7 Fig The effect of aging and stress on *TERRA* and H3K9me3. (A) *TERRA* Northern blots on RNA isolated from zebrafish liver at ZT2 and ZT10 under different conditions (Adult; 12M, Old; 20M, stressed n = 2). (B) The level of H3K9me3 at the subtelomere of chromosome 1 from zebrafish liver was measured by ChIP for 3 different age groups (Young; 4M, Adult; 12M, Old; 20M). (C)Same as in B except the tissue was skeletal muscle. The data in B &C represent the average of 3 independent biological replicates. The error bars represent the SEM and analysis was by student t-test (*; p ≤ 0.05).
(EPS)
