# Supplementary material for: BMAL1 associates with chromosome ends to control rhythms in TERRA and telomeric heterochromatin
Source: PLoS One. 2019 Oct 21;14(10):e0223803. doi: 10.1371/journal.pone.0223803 (PMC6802832; doi:10.1371/journal.pone.0223803)
Supplement: S1 Table — (DOCX) [file pone.0223803.s001.docx]

**S1 Table: Oligonucleotides**

| Tel4L_800 F | CCTACTTTCCTATCCCGTTTTCC |
| --- | --- |
| Tel4L_800 R | GGGCGGAGATAAATATAAATAGAATAAAGA |
| Nc_CboxF | TTGGCCGGACAACCAGTAC |
| Nc_CboxR | AGCCTCCAGATCTCAATTTTGC |
| Telomere_F | GGTTTTTGAGGGTGAGGGTGAGGGTGAGGGTGAGGGT |
| Telomere_R | TCCCGACTATCCCTATCCCTATCCCTATCCCTATCCCTA |
| Zf_Subtelo_F | TGTGTTCGTGGAGTGGATCTTG |
| Zf_Subtelo_R | GCGTGATCGTGGTTATGTTCA |
| Zf_Ebox_F | CCATGCAAGCTATTGTAAAAGCA |
| Zf_Ebox_R | TGTGGTCTCGCCCTGTTTG |
| mDbp_F | TGGGACGCCTGGGTACAC |
| mDbp_R | GGGAATGTGCAGCACTGGTT |
| mTelomere_chr13_F | CAAGTGTCCCCAGGAGATGT |
| mTelomere_chr13_R | CCACCATCACAGGTCACAAG |
| m_Per2_F | CACGCTGGCAACCTTGAAGT |
| m_Per2_R | TGGTAGTACTCCTCATTAGCCTTCAC |
| m_Bmal1_F | GATCGAAAAAGCTTCTGCACAA |
| m_Bmal1_R | GGGTGGCCAGCTTTTCAA |
| m_eif2a_F | GCTGGGACGCCTAACCTACA |
| m_eif2a_R | GGATGAACGATTTCAAACATGCT |
| pTelo250F | ATTCGCCCTTCCCTAACC |
| pTelo250R | TTCGCCCTTGTTAGGGTT |
